# Subgaleal Effusion and Brain Midline Shift After Cranioplasty: A Retrospective Study Between Polyetheretherketone Cranioplasty and Titanium Cranioplasty After Decompressive Craniectomy

**DOI:** 10.3389/fsurg.2022.923987

**Published:** 2022-07-21

**Authors:** Tao Ji, Peiwen Yao, Yu Zeng, Zhouqi Qian, Ke Wang, Liang Gao

**Affiliations:** ^1^ Department of Neurosurgery, Shanghai Tenth People's Hospital, Tongji University, Shanghai, China; ^2^ School of Clinical Medicine, Nanjing Medical University, Nanjing, China

**Keywords:** cranioplasty, decompressive craniectomy, PEEK, titanium mesh, subgaleal effusion, brain midline shift

## Abstract

Cranioplasty with polyetheretherketone (PEEK) has recently shown better cerebral protection performance, improved brain function, and aesthetic contour compared with titanium mesh. However, whether patients undergoing PEEK cranioplasty tend to develop subgaleal effusions remains elusive. This retrospective study included patients who underwent cranioplasty with PEEK implants or titanium mesh after decompressive craniectomy between July 2017 and July 2020. Patient information, including general information, location, size of the defect, subgaleal depth, and brain midline shift was collected and statistically analyzed. There were 130 cases of cranioplasty, including 35 with PEEK implants and 95 with a titanium mesh. Patients who underwent cranioplasty with a PEEK implant had a higher subgaleal effusion rate than those who underwent cranioplasty with titanium mesh (85.71% vs. 53.68%, *P* < 0.001), while a midline shift >5 mm was more frequently observed in the PEEK group than in the titanium group (20% vs. 6.3%, *P* = 0.021). The PEEK material was the only factor associated with subgaleal effusion after cranioplasty (OR 5.589, *P* = 0.002). Logistic regression analysis further showed that age was a protective factor against midline shift in the PEEK cranioplasty group (OR 0.837, *P* = 0.029). Patients who underwent cranioplasty with PEEK implants were more likely to develop severe subgaleal effusion and significant brain midline shifts than those with titanium mesh implants.

## Introduction

Cranioplasty is most commonly performed after a previous craniectomy for traumatic brain injury, stroke, intracranial tumour resection, or other aetiologies, which provides a combination of cerebral protection and aesthetic improvement. Autologous bone flaps and bone grafts are the most commonly used implant materials because of their biocompatibility. However, several pitfalls, including difficulties with long-term preservation (1), high infection rate (2), and potential autolysis (3), outweigh its benefits. In addition, autologous bone flaps may also be unavailable because of previous infections and traumatic damage (4, 5). Therefore, synthetic alloplastic materials, including metallics, acrylics, ceramics, and plastics, have been considered as alternatives.

Titanium mesh is a widely used metallic implant in cranioplasty because of its non-corrosive properties and high overall strength (6). However, post-operative seizures are a common concern, leading to reoperation and implant removal. In recent decades, patient-specific implants with polymeric materials, including hydroxyapatite, polymethyl methacrylate (PMMA), and polyetheretherketone (PEEK), have been used instead of titanium mesh (7–9). In the long term, hydroxyapatite has shown a lower complication rate and better osseointegration effect in clinical use (10). Computer-aided design and manufacturing (CAD/CAM) technology enables the pre-operative prefabrication of polymeric materials and allows precise intra-operative time-saving reconstruction (11).

The polyaromatic semicrystalline polymer, PEEK, is commonly used as an implant material in spine reconstruction (12–14) and has been used in craniofacial reconstruction in recent decades (9, 15–17). Furthermore, PEEK is thermostable in the human body (18) and compatible with CT and MRI as it does not result in artefact formation (19). Compared with titanium mesh, it can provide a more aesthetic skull contour, adequate cerebral protection, and satisfactory imaging compatibility, with fewer complications (15, 20–23). Compared with other polymeric materials, PEEK showed elasticity and tensile properties that mimic human bone and provided better protection during lab experiments (24).

Cranioplasty was associated with significant complications (25). During clinical practise, we found that patients who underwent PEEK cranioplasty following decompressive craniectomy tended to develop subgaleal effusion, compared with those who underwent titanium mesh cranioplasty, generally in the first week after cranioplasty. Although several studies have compared the outcomes of titanium mesh and PEEK cranioplasties, subgaleal effusion has rarely been discussed. The present study is the first to retrospectively analyze the subgaleal effusion rate among patients who underwent cranioplasty following decompressive craniectomy with PEEK or titanium mesh during hospitalzsation and to discuss the management of this short-term complication of PEEK cranioplasty.

## Methods and Materials

### Collection of Clinical Data

We performed a retrospective review of patients who underwent cranioplasty after decompressive craniectomy with complete clinical data between July 2017 and July 2020 at the Neurosurgery Department of Shanghai Tenth People's Hospital. General information, indication, location, size of the defect, maximum depth of subgaleal effusion, offset distance of midline, and other complications were extracted from the medical records. Written informed consent was obtained from all patients. Before surgery, a presurgical discussion with radiologists and anaesthesiologists was performed. Both PEEK and titanium cranioplasties were performed by three experienced neurosurgeons in our department. This study was approved by the Institutional Review Board of Shanghai Tenth People's Hospital (No. 051219019).

### Statistical Analysis

Statistical analyses were performed using Prism version 8.0 (GraphPad, USA) and SPSS Statistics 25 (IBM, USA). Continuous data were presented as mean ± standard deviation (SD) and compared using the Student's t-test or Mann–Whitney U test, as appropriate. Frequency data were compared using Fisher's exact test or chi-square test, as appropriate. Binary logistic regression analysis was performed to determine associated factors. All tests were two-tailed. Statistical significance was set at *P* < 0.05.

## Results

### Patient Information

The clinical characteristics of the patients are summarised in Table 1. A total of 130 cranioplasty procedures were performed, with PEEK implants in 35 cases and titanium mesh in 95 cases. Porous PEEK scaffolds with 3D pore sizes of 4 mm placed 1 cm apart were used for all PEEK cranioplasties. In the PEEK group, there were 23 male (65.71%) and 12 female patients (34.29%), with a mean age of 40.31 ± 15.47 years; in the titanium mesh group, there were 69 male (72.63%) and 26 female patients (27.37%), with a mean age of 47.25 ± 13.85 years. The median interval between the last decompressive craniectomy and cranioplasty was 5.20 ± 2.68 months in the PEEK group and 5.17 ± 3.12 months in the titanium group. There were 30 (85.71%) unilateral and 5 (14.29%) bilateral skull defects in the PEEK group, with a median bony defect measured at 73.87 ± 27.10 cm^2^ (interquartile range, 59.93–86.51 cm^2^), while there were 85 (89.47%) unilateral and 10 (10.53%) bilateral skull defects in the titanium mesh group, with a bony defect measuring 83.55 ± 26.04 cm^2^ (Table 1).

**Table 1:** Clinical characteristics of patients who underwent cranioplasty.

	Materials		*P* value
	PEEK group (*N* = 35)	Titanium group (*N* = 95)	
Gender, *n*(%)			
Males	23(65.71)	69(72.63)	0.442
Females	12(34.29)	26(27.37)	
Mean Age ± SD, years	40.31 ± 15.47	47.25 ± 13.85	0.015
Mean Interval ± SD, months	5.20 ± 2.68	5.17 ± 3.12	0.960
Bilateral, *n*(%)			
Yes	5(14.29)	10(10.53)	0.552
No	30(85.71)	85(89.47)	
Mean Defect size ± SD, cm^2^	73.87 ± 27.10	83.55 ± 26.04	0.065

### PEEK Material is the Only Significant Factor Associated with Subgaleal Effusion After Cranioplasty

A subgaleal drainage tube was placed in all patients and usually removed on day two after cranioplasty. For post-operative evaluation, patients underwent routine cranial CT. In cases of subgaleal effusion >10 mm or brain midline shift >5 mm, subgaleal puncture and drainage were performed, followed by a compression bandage.

Subgaleal effusion occurred in 30 patients who underwent PEEK cranioplasty (85.71%), whereas only 53.68% (51/95) of patients in the titanium mesh group developed subgaleal effusion. The median drainage volume on the first day after cranioplasty was 165.00 ± 83.94 ml and 152.90 ± 65.87 ml in the PEEK and titanium groups, respectively (*P* = 0.393). The subgaleal effusion depth was 6.30 ± 3.72 mm in the PEEK group compared with that in the titanium mesh group (6.79 ± 8.97 mm (*P* = 0.044, Table 2). Further, logistic regression showed that the PEEK material was the only significant factor associated with subgaleal effusion (Odds ratio (OR), 5.589; 95% confidence interval (CI), 1.90–16.46; *P* = 0.002, Table 3).

**Table 2:** Subgaleal effusion and brain midline shift in the PEEK and Titanium groups.

	PEEK group	Titanium mesh group	*P* value
Subgaleal effusion, *n*(%)			<0.001
Yes	30(85.71)	51(53.68)	
No	5(14.29)	44(46.32)	
Mean drainage volume on the first day after cranioplasty ± SD, mL	165.00 ± 83.94	152.90 ± 65.87	0.393
Mean effusion Depth ± SD, mm	6.30 ± 3.72	6.79 ± 8.97	0.044
Brain midline shift, *n*(%)			0.021
<5 mm	28(80)	89(93.68)	
>5 mm	7(20)	6(6.32)	

**Table 3:** Binary logistic regression analysis for factors associated with subgaleal effusion.

	OR(95% CI)	*P* value
Gender		
Male	1	
Female	1.525(0.636–3.657)	0.344
Age	1.012(0.984–1.041)	0.392
Interval	0.975(0.856–1.110)	0.702
Defect Size	1.001(0.985–1.017)	0.919
Bilateral		
Yes	1	
No	0.894(0.260–3.078)	0.823
Drainge volume	1.001(0.995–1.007)	0.681
Material		
Titanium	1	
PEEK	5.588567(1.90–16.46)	0.02

### Age is a Protective Factor Against Brain Midline Shift in the PEEK Cranioplasty Group

Notably, a brain midline shift of >5 mm was more frequently observed in the PEEK group, with an incidence of 20% (7/35), than in the titanium group, with an incidence of 6.32% (6/95) (*P* = 0.021, Table 2). Intriguingly, age was a significant factor associated with less brain midline shift in the PEEK cranioplasty group (OR 0.837, 95% CI 0.713–0.982, *P* = 0.029), whereas effusion depth was not (OR 1.041, 95% CI 0.901–1.202, *P* = 0.589; Table 4).

**Table 4:** Binary logistic regression analysis for factors associated with brain midline shift in the PEEK cranioplasty group.

	OR(95% CI)	*P* value
Gender		
Male	1	
Female	7.801(0.576–105.628)	0.122
Age	0.837(0.713–0.982)	0.029
Interval	1.486(0.787–2.805)	0.222
Defect Size	0.965(0.920–1.012)	0.145
Bilateral		
Yes	1	
No	3.381(0.083–137.515)	0.519
Effusion depth	1.041(0.901–1.202)	0.589

### Presentation of a Typical Vignette

**Figure 1:**
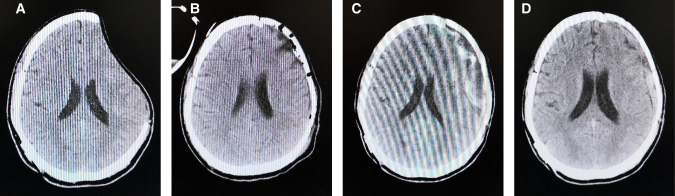
CT scan images before PEEK cranioplasty (**A**), right after the operation (**B**), day five post-operatively (**C**), and day ten post-operatively (**D**).

A 29-year-old man went into a progressive coma due to a traffic accident and underwent decompressive cranioplasty 3 months before admission to our hospital. Physical examination found a 90.22 cm^2^ defect in the left frontal–parietal region (Figure 1A). A PEEK implant was implanted and the operation was uncomplicated (Figure 1B). In the following days, a routine CT showed that the patient developed subgaleal effusion, which reached a maximum of 21.4 mm on day five after PEEK cranioplasty (Figure 1C), while no other complications were observed. Subgaleal puncture was performed. On day ten postoperatively, CT showed that most of the effusion had been eliminated (Figure 1D) and the patient was discharged without complications. During the post-operative follow-up period, the patient recovered well.

## Discussion

Cranioplasty is associated with significant complications (25), and a consensus on the ideal alloplastic material remains elusive. A fresh autologous graft is the first choice for cranioplasty because of its ideal structure and osteogenic potential (26). However, high infection and exposure rates have been observed in patients implanted with autologous grafts, especially in those with repeated reconstruction (27). Moreover, the self-resorption of bone grafts is another concern. Bone flap resorption is more likely to occur in young patients (≤18 years) and in patients with a history of decompressive craniectomy due to traumatic brain injury (28). For these patients, alloplastic implants such as titanium and PEEK would be an alternative for cranioplasty. However, these materials are far from the final answer, in which problems are emerging successively. A prospective multicentre cohort study spanning over 6 months in the United Kingdom (UK) and Ireland showed that titanium mesh remained the most commonly used material for cranioplasty, representing 64% of all cranioplasties (29). Further investigation of the properties of other cranioplasty materials, such as hydroxyapatite and PEEK, may facilitate the selection of optimal materials for cranioplasty.

Titanium is relatively cost-saving, and reduced operation time and intra-operative haemorrhage have been observed during covered cranioplasty with a titanium mesh (30) since the skull edge is not separated. However, a higher infection rate was found in patients with a history of radiotherapy who underwent titanium cranioplasty (15). Future exposure of the titanium mesh is another long-term complication with an incidence of 14% (22), necessitating reoperation and removal of the implant. Nevertheless, titanium mesh may cause artefacts during CT or MRI (31). The PMMA family behaves in a brittle manner under strong impact forces, is exothermic during polymerisation, and may cause further damage to the surrounding tissues (32). A single-centre cohort study provided Level 3 evidence that custom-made hydroxyapatite bone flaps showed better osseointegration, lower reoperation rate, and higher patient satisfaction than PMMA materials (33). However, they still fail to provide sufficient protection against blunt trauma and are prone to fracture (30).

Therefore, PEEK seems to be a more appropriate choice than other alloplastic materials for calvarial reconstruction because of the reduced infection and donor site morbidity, sufficient tensile property, and aesthetic contour seen in PEEK cranioplasty (21, 22, 34–36). The convex shape of CT-modeled implants, such as PEEK implants, could ensure sufficient space for the brain parenchyma to expand. Still, in cases of prolonged defect or repeated surgeries, the brain parenchyma may expand slowly or insufficiently, and the dead space exists, leading to further formation of subgaleal effusion or even abcesses (37, 38).

In accordance with this, our results showed that subgaleal effusion brain midline shift was more likely to develop in patients who underwent PEEK cranioplasty within the first week after surgery. Among several clinical factors, including sex, age, interval, defect size, and material type, PEEK was the only predictor of subgaleal effusion. Of note, individual surgical nuances such as the type of dural closure may affect the rate of subgaleal fluid collection (39). The dura was carefully protected during our surgery, and a clear inspection of the dura for potential CSF leakage was routinely performed at the end of the surgery. We observed a limited number of cases of dural tears during both PEEK and titanium cranioplasty. In this case, a closed suture with a watertightness test was performed to ensure dural closure. Moreover, in the PEEK cranioplasty group, age was a significant predictor of a midline shift in the brain. This is, to some extent, due to the variable mass effect depending on the volume of fluid collection and the degree of atrophy (40), as younger patients with less atrophy may face a greater risk of mass effect. Therefore, a close monitoring of younger patients undergoing PEEK cranioplasty is necessary.

In the case of subgaleal effusion, a timely subgaleal puncture could provide satisfactory management, and none of the patients underwent secondary surgery. It is suggested that vascularised tissue coverage of the implants during cranioplasty may be a safe and effective way to prevent subgaleal effusion by minimising the subgaleal dead-space (38, 41). Although subgaleal drainage can reduce subgaleal fluid collection, it may also induce wound infections, intracranial hypotension, or even the infectious destruction of anatomical structures (42). A recent retrospective study investigating the correlation between subgaleal effusion and intracranial infection after autologous cranioplasty found that placement of a subgaleal drain is a protective factor against intracranial infection, and no significant difference in the length of drainage was observed between those with and those without (43). However, the drainage persisted for 3 days on average in this study, and whether prolonged drainage (e.g., within one week) is associated with increased intracranial infection remains elusive. Of note, two ongoing clinical trials investigated subgaleal effusion in patients who underwent PEEK cranioplasty or titanium cranioplasty (44, 45), which may provide further evidence for this common but rarely studied complication of cranioplasty.

Our study has several limitations. First, the present study focused on short-term complications during hospitalisation, and extended follow-up is needed to determine the correlation between subgaleal effusion and long-term outcomes, including mortality rates, cerebral blood flow, and cognitive function. The second limitation was the retrospective nature of this study. Future prospective studies on larger patient cohorts may provide more reliable evidence for this phenomenon. Third, our study is not sufficient to conclude the relationship between the properties of PEEK and the higher incidence of epidural effusion. Insights into the biomechanics of PEEK, surface design patterns, and communication between subgaleal and extradural/subdural spaces may further facilitate the choice of cranioplasty material (46). Finally, the timing of cranioplasty has a certain impact on patients' outcomes (47), but this issue is not discussed in the present study, as this is a small cohort. Further subgroup analyses of subgaleal effusion based on the timing of cranioplasty will provide a better understanding of the relationship between the timing of surgery and this poorly understood complication.

## Conclusions

Although several studies have compared the outcomes of titanium and PEEK cranioplasty, subgaleal effusion has not been thoroughly investigated. The present study described a single-institution observation of subgaleal effusion as a short-term complication that was more frequently observed in patients who underwent PEEK cranioplasty than in those who underwent titanium cranioplasty. Routine postoperative CT is suggested within the first week after PEEK cranioplasty, and subgaleal puncture can be performed if needed. Future multicentre, randomised controlled trials focusing on subgaleal effusion after cranioplasty may provide more solid evidence.

## Data Availability

The raw data supporting the conclusions of this article will be made available by the authors, without undue reservation.
